# Association of genetic polymorphisms in *CASP7* with risk of ischaemic stroke

**DOI:** 10.1038/s41598-019-55201-y

**Published:** 2019-12-09

**Authors:** Zhaoshi Zheng, Songyan Liu, Chuheng Wang, Chunhui Wang, Dong Tang, Yuqing Shi, Xuemei Han

**Affiliations:** 1 No. 1 Department of Neurology, China-Japan Union Hospital of Jilin University, Changchun, Jilin, 130031 P.R. China; 2 Department of Clinical Medicine (Grade 2017 Student), School of Basic Medicine, Zhengzhou University, Zhengzhou, Henan 450001 P.R. China; 3 Department of Neurosurgery, the Hospital of Jilin Province, Changchun, Jilin, 130031 P.R. China

**Keywords:** Genetics of the nervous system, Risk factors

## Abstract

Caspase 7 (*CASP7*) is located on chromosome 10q25.3 that has been identified to be a susceptibility locus of ischaemic stroke (IS) by genome-wide association study. Elevated *CASP7* was observed in IS, acting as a key apoptotic mediator in the development of IS. The aim of this study was to investigate the association between genetic polymorphisms in *CASP7* and risk of IS. The *CASP7* polymorphisms were genotyped using a TaqMan allelic discrimination assay. The expression levels of *CASP7* mRNA were examined using quantitative polymerase chain reaction and luciferase activity was analyzed using the Dual Luciferase reporter assay. The rs12415607 in the promoter of *CASP7* was associated with a reduced risk of IS (AA vs. CC: adjusted OR = 0.55, 95% CI: 0.38–0.80, *P* = 0.002; CA/AA vs. CC: adjusted OR = 0.70, 95% CI: 0.54–0.91, *P* = 0.007; AA vs. CC/CA: adjusted OR = 0.64, 95% CI: 0.46–0.90, *P* = 0.01; A vs. C: adjusted OR = 0.74, 95% CI: 0.62–0.89, *P* = 0.001). Moreover, the rs12415607 AA genotype carriers exhibited lower levels of *CASP7* mRNA and the rs12415607 A allele decreased the promoter activity. These findings indicate that the rs12415607 A allele induces lower levels of transcriptional activity and *CASP7* mRNA, and thus is associated with a reduced risk of IS.

## Introduction

Cerebral ischaemia may lead to stroke, which is a main reason for mortality and permanent disability in both developed and developing countries^1,2^. Among all, ischaemic stroke (IS) accounts for about 85–90% of the stroke cases^3,4^. Although the exact etiology is not full known, conventional risk factors have been identified, such as hypertension, diabetes mellitus, and dyslipidemia^5–8^. Additionally, previous genetic epidemiological studies provided substantial evidence that single nucleotide polymorphisms (SNPs) may be involved in the development of IS. For example, we previously reported that long non-coding RNA growth arrest-specific 5 (GAS5) rs145204276 ins/ins genotype and miR-181b rs322931 CT and CT/TT genotypes were associated with an increased risk of IS^9,10^.

Ischaemia preferentially triggers neuronal damage through an apoptotic-like process rather than necrosis^11^. Caspases, a family of cysteine aspartases, play a critical role in apoptotic cell death, including delayed neuronal death following IS^11,12^. It is evident that caspases are cleaved and activated in human brains and experimental models of stroke and neurodegenerative diseases^13–15^. When activated, executioner caspases (caspases 3 and 7) facilitate cell demise by targeting and degrading numerous substrate proteins^11^. Cell death can be attenuated by administering endogenous caspase inhibitors during and after brain ischaemic injury^16–18^. The neuroprotective role was also observed in caspase 3 deficient mice that were more resistant to ischaemic stress both *in vivo* and *in vitro*^19^. These findings suggest that drugs targeting caspase-independent programmed cell death may be an effective therapy for IS.

Since 2007, genome-wide association study (GWAS) has been used in exploring susceptibility genes of human diseases. To date, several risk loci of IS have been identified, such as chromosome 10q25, 12p13, 14q13.3, 1p13.2, 12q24.12, 10q11.21, 9p21, and 1p32^20–27^. Caspase 7 (*CASP7*), located on chromosome 10q25.3, has been reported to be upregulated in a rat model of focal cerebral ischemia^28^, acting as a key apoptotic mediator in the development of IS. Based on this background, we hypothesized that SNPs in *CASP7* may affect the risk of IS. To test this hypothesis, we carried out a case-control study to evaluate the role of SNPs in *CASP7* in the development of IS in a Chinese population. Genotype-phenotype and potential mechanism analysis was also explored.

## Materials and Methods

### Study population

The study protocol was approved by the Institutional Review Board of the China-Japan Union Hospital of Jilin University, and informed consent was obtained from all individual participants included in the study. The characteristics of the study population have been described previously^9,10^. Briefly, 505 patients with IS and 652 controls were consecutively obtained from the China-Japan Union Hospital of Jilin University between March 2014 and July 2017. IS diagnosis was confirmed based on clinical manifestations and computed tomography scans and/or magnetic resonance imaging. Patients were excluded if they had the following medical records: hemorrhagic stroke, subarachnoid hemorrhage, traumatic brain injury, malignancy, and brain inflammatory diseases. The controls were enrolled from the same hospital during the same time period if they were healthy Chinese Han subjects and did not report any family history of IS and thromboembolic diseases. The controls were frequency matched to cases based on age, gender, ethnicity, and living area. Clinical information was collected from medical records, including age, gender, hypertension, diabetes mellitus, total cholesterol (TCH), triglyceride (TG), high-density lipoprotein cholesterol (HDL-C), and low-density lipoprotein cholesterol (LDL-C).

### SNPs selection

We selected SNPs in *CASP7* according to the following criteria: (1) TagSNPs; (2) Minor allele frequency more than 10% in Chinese Han population; and (3) Bioinformational analysis predicted to be functional. Finally, 5 SNPs were identified, including a nonsynonymous polymorphism in exon 8 of *CASP7* (rs2227310), a polymorphism in the promoter region of *CASP7* (rs12415607) with the C but not the A allele creating a binding site to the transcriptional factor TFII-I, and 3 polymorphisms in the 3′-untranslated region (3′-UTR) of *CASP7* with different allele having different affinity to miRNAs. Detailed information of the 3 polymorphisms in the 3′-UTR of *CASP7* is presented in Supplementary Table 1.

### DNA extraction and genotyping

Genomic DNA was extracted from whole blood using a commercial kit from Tiangen company (Beijing, China). Genotyping was performed using a TaqMan allelic discrimination assay (Applied Biosystems, Foster City, CA, USA). Polymerase chain reaction (PCR) was run on the ABI 7500 real-time PCR System (Applied Biosystems) using the following probes for the 5 polymorphisms: C__27432681_20, C__500779_20, C__500776_10, C__12119563_10, and C__500777_10, respectively. For quality control, negative control in each run was performed using distilled water as template and accuracy of genotyping was confirmed by DNA sequencing.

### RNA isolation and quantitative real-time PCR (qPCR)

Total cellular RNA was extracted from peripheral blood cells using the RNAprep pure Blood Kit (Tiangen, Beijing, China) according to the manufacturer’s instructions. A total of 1 μg RNA was reverse-transcribed using the First Strand cDNA Synthesis Kit (Thermo Fisher Scientific, Waltham, MA, USA) according to the manufacturer’s instructions. Relative expression levels of *CASP7* were determined by qPCR using the following primers: *CASP7* sense primer: CGTTTGTACCGTCCCTCTTC and antisense primer: GCCCAGCTTTTCAAAATTCA; *GAPDH* sense primer: CTCTCTGCTCCTCCTGTTCG AC and antisense primer: TGAGCGATGTGGCTCGGCT. Each 10 μL qPCR reaction contained 5 μL of 2 X SYBR Green master mix (Thermo Fisher Scientific), 10 μM each primer and 1 μL of cDNA. PCR conditions were set as follows: 95 °C for 2 min, 40 cycles of 95 °C for 15 sec and 60 °C for 1 min. The relative expression levels of *CASP7* mRNA were calculated using the 2^−ΔΔCt^ method^29^.

### Plasmid constructs

Genomic DNA from human *CASP7* promoter region was amplified by PCR using primers: 5′-TTCTCGAGAAAAGACTAGGGCAGCCACA-3′ (forward) and 5′-CAAGCTTGCCCCTCGCTCTACAAAGTT-3′ (reverse). For constructing luciferase reporter plasmids, oligonucleotides containing the rs12415607 A and C allele were cloned into pGL3-basic vector (Promega, Madison, WI, USA) after digestion with *Xho* I and *Hind* III. All PCR products were verified by DNA sequencing.

### Cell culture and dual luciferase reporter assay

Human embryonic kidney (HEK293) cells were cultured in Dulbecco’s modified Eagle medium supplemented with 10% FBS (HyClone, Logan, UT, USA) and 1% penicillin-streptomycin (Life Technologies, Grand Island, NY, USA). pGL3-rs12415607A, pGL3-rs12415607C, and pGL3 empty vector (1 μg) were introduced into cells per well in a 12-well plate using Lipofectamine 3000 (Thermo Fisher Scientific) according to the manufacturer’s protocol. Renilla luciferase pRL vector (20 ng) was cotransfected as an internal control. Luciferase activities were checked at 48 h after transfection using the dual luciferase reporter assay system (Promega) and relative luciferase activities were measured using the ratio of firefly luciferase activity to renilla luciferase activity.

### Statistical analysis

Continuous data were reported as mean ± standard deviation and compared using Student’s *t* test. Dichotomous data were reported as frequencies (percentages) and compared using χ^2^ test. Hardy-Weinberg equilibrium (HWE) and the association of the 5 SNPs with IS risk were evaluated using χ^2^ test. Odds ratios (ORs) and 95% confidence intervals (CIs) were computed after adjustment for age, gender, hypertension, and diabetes mellitus. Bonferroni corrected test was used for multiple comparisons and the corrected *P* value was set as 0.01 (0.05/5). Binary logistic regression was used to identify independent risk factors for IS. Haplotype analysis was performed using a SHEsis software (http://analysis.bio-x.cn/myAnalysis.php)^30^. Relative expression levels of *CASP7* mRNA were reported as median with interquartile range and compared using Whitney U test. Statistics were performed using the SPSS software version 19.0 (SPSS, Chicago, IL, USA) and a two-sided *P* < 0.05 was considered significant.

### Ethics approval

All procedures performed in studies involving human participants were in accordance with the ethical standards of China-Japan Union Hospital of Jilin University and with the 1964 Helsinki declaration and its later amendments or comparable ethical standards.

## Results

### Characteristics of study population

Table 1 shows the characteristics of the study population. The mean age of the patients was 59.9 ± 10.9 years, which was similar to that of the controls (59.0 ± 11.9 years; *P* = 0.16 for *t-*test). Of the 505 patients, 325 (64.4%) were males vs. 61.2% of controls (*P* = 0.27 for χ^2^ test). The percentage of hypertension and diabetes mellitus in IS patients was significantly higher than that in controls (*P* < 0.001 and = 0.01, respectively). The serum levels of TCH, TG, and LDL-C in IS patients were higher than controls (*P* < 0.001), while there was no difference of HDL-C levels between cases and controls (*P* = 0.43).

**Table 1:** Characteristics of the study population.

Variables	Controls, n = 652	Patients with IS, n = 505	*P* value
Age, mean (±SD)	59.0 (±11.9)	59.9 (±10.9)	0.16
Gender (%)			
Male	399 (61.2)	325 (64.4)	0.27
Female	253 (38.8)	180 (35.6)	
Hypertension, n (%)			
Yes	132 (20.2)	277 (54.9)	<0.001
No	520 (79.8)	228 (45.1)	
Diabetes mellitus, n (%)			
Yes	70 (10.7)	79 (15.6)	0.01
No	582 (89.3)	426 (84.4)	
TCH, mmol/L	4.69 ± 0.79	5.04 ± 0.72	<0.001
TG, mmol/L	1.11 ± 0.36	1.85 ± 1.10	<0.001
HDL-C, mmol/L	1.55 ± 0.36	1.57 ± 0.38	0.43
LDL-C, mmol/L	2.26 ± 0.97	2.68 ± 0.98	<0.001

IS, ischemic stroke; SD, standard deviation; TCH, total cholesterol; TG: triglyceride; HDL-C, high-density lipoprotein cholesterol; LDL-C, low-density lipoprotein cholesterol.

### Association between *CASP7* polymorphisms and risk of IS

The genotype distributions of the 5 SNPs were in HWE among controls (*P* > 0.05). The rs12415607 in the promoter of *CASP7* was associated with a reduced risk of IS in homozygote comparison, dominant comparison, recessive comparison, and allele comparison (AA vs. CC: adjusted OR = 0.55, 95% CI: 0.38–0.80, *P* = 0.002; CA/AA vs. CC: adjusted OR = 0.70, 95% CI: 0.54–0.91, *P* = 0.007; AA vs. CC/CA: adjusted OR = 0.64, 95% CI: 0.46–0.90, *P* = 0.01; A vs. C: adjusted OR = 0.74, 95% CI: 0.62–0.89, *P* = 0.001). However, the rs2227310, rs10787498, rs1127687, and rs4353229 were not associated with the risk of IS (Table 2).

**Table 2:** Association between *CASP7* polymorphisms and risk of IS.

Polymorphisms	Controls, n = 652 (%)	IS, n = 505 (%)	Adjusted OR (95% CI)^†^	*P* value
rs12415607				
CC	219 (33.6)	215 (42.6)	1.00	
CA	300 (46.0)	223 (44.2)	0.76 (0.58–1.00)	0.05
AA	133 (20.4)	67 (13.3)	0.55 (0.38–0.80)	0.002
Dominant model	433 (66.4)	290 (57.4)	0.70 (0.54–0.91)	0.007
Recessive model	519 (79.6)	438 (86.7)	0.64 (0.46–0.90)	0.01
C allele	738 (56.6)	653 (64.7)	1.00	
A allele	566 (43.4)	357 (35.3)	0.74 (0.62–0.89)	0.001
rs2227310				
CC	216 (33.1)	191 (37.8)	1.00	
CG	334 (51.2)	243 (48.1)	0.84 (0.64–1.10)	0.21
GG	102 (15.6)	71 (14.1)	0.85 (0.58–1.24)	0.39
Dominant model	436 (66.9)	314 (62.2)	0.84 (0.65–1.10)	0.21
Recessive model	550 (84.4)	434 (85.9)	0.94 (0.66–1.33)	0.71
C allele	766 (58.7)	625 (61.9)	1.00	
G allele	538 (41.3)	385 (38.1)	0.91 (0.76–1.09)	0.29
rs10787498				
TT	369 (56.6)	298 (59.0)	1.00	
TG	253 (38.8)	189 (37.4)	0.89 (0.69–1.16)	0.39
GG	30 (4.6)	18 (3.6)	0.74 (0.39–1.41)	0.35
Dominant model	283 (43.4)	207 (41.0)	0.88 (0.68–1.13)	0.30
Recessive model	622 (95.4)	487 (96.4)	0.77 (0.41–1.47)	0.43
T allele	991 (76.0)	785 (77.7)	1.00	
G allele	313 (24.0)	225 (22.3)	0.89 (0.72–1.09)	0.26
rs1127687				
GG	430 (66.0)	325 (64.4)	1.00	
AG	198 (30.4)	165 (32.7)	1.11 (0.84–1.45)	0.46
AA	24 (3.7)	15 (3.0)	0.73 (0.35–1.50)	0.39
Dominant model	222 (34.0)	180 (35.6)	1.07 (0.82–1.38)	0.63
Recessive model	628 (96.3)	490 (97.0)	0.72 (0.35–1.46)	0.36
G allele	1058 (81.1)	815 (80.7)	1.00	
A allele	246 (18.9)	195 (19.3)	1.01 (0.81–1.27)	0.91
rs4353229				
TT	205 (31.4)	155 (30.7)	1.00	
CT	320 (49.1)	260 (51.5)	1.11 (0.84–1.48)	0.45
CC	127 (19.5)	90 (17.8)	0.92 (0.63–1.33)	0.65
Dominant model	447 (68.6)	350 (69.3)	1.07 (0.81–1.40)	0.64
Recessive model	525 (80.5)	415 (82.2)	0.87 (0.63–1.20)	0.39
T allele	730 (56.0)	570 (56.4)	1.00	
C allele	574 (44.0)	440 (43.6)	0.99 (0.82–1.18)	0.87

*CASP7*, caspase 7; IS, ischemic stroke; OR, odds ratio; CI, confidence interval.

^†^Adjusted by age, gender, hypertension, and diabetes mellitus.

### Haplotype analysis

Nine common haplotypes are summarized in Table 3. Compared to the CCTGT haplotype, the CCGAT and AGGAT haplotypes were associated with a reduced risk of IS (CCGAT vs. CCTGT: OR = 0.40, 95% CI: 0.24–0.67, *P* = 3.34E-4; AGGAT vs. CCTGT: OR = 0.38, 95% CI: 0.20–0.73, *P* = 0.003, respectively), whereas the CCGGC, CCGGT, and AGGGT haplotypes were associated with an increased risk of IS (CCGGC vs. CCTGT: OR = 5.86, 95% CI: 3.39–10.12, *P* = 4.74E-12; CCGGT vs. CCTGT: OR = 5.66, 95% CI: 3.18–10.09, *P* = 1.43E-10; AGGGT vs. CCTGT: OR = 2.19, 95% CI: 1.23–3.92, *P* = 0.007, respectively).

**Table 3:** Haplotype analysis of *CASP7* polymorphisms with risk of IS.

Haplotypes^†^	Controls (%)	IS (%)	OR (95% CI)	*P* value
CCTGT	309 (23.7)	208 (20.6)	1.00	
CCTGC	260 (19.9)	170 (16.8)	0.97 (0.75–1.26)	0.83
AGTGT	233 (17.9)	134 (13.3)	0.85 (0.65–1.13)	0.26
AGTGC	166 (12.7)	94 (9.3)	0.84 (0.62–1.15)	0.27
CCGAT	78 (6.0)	21 (2.1)	0.40 (0.24–0.67)	3.34E-4
CCGGC	18 (1.4)	71 (7.0)	5.86 (3.39–10.12)	4.74E-12
CCGGT	16 (1.2)	61 (6.0)	5.66 (3.18–10.09)	1.43E-10
AGGAT	47 (3.6)	12 (1.2)	0.38 (0.20–0.73)	0.003
AGGGT	21 (1.6)	31 (3.1)	2.19 (1.23–3.92)	0.007

*CASP7*, caspase 7; IS, ischemic stroke; OR, odds ratio; CI, confidence interval.

^†^Only the frequency more than 1% was presented.

### Multivariate regression analysis

Multivariate logistic regression analysis was performed. As shown in Table 4, 6 independent risk factors of IS were identified, that is, hypertension (OR = 4.62, 95%CI: 3.39–6.30, *P* = 3.86E-22), TCH (OR = 1.24, 95%CI: 1.02–1.50, *P* = 0.03), TG (OR = 6.10, 95%CI: 4.55–8.20, *P* = 2.66E-33), LDL-C (OR = 1.95, 95%CI: 1.65–2.31, *P* = 8.00E-15), rs12415607 (OR = 13.67, 95%CI: 3.77–49.48, *P* = 6.81E-5), and rs2227310 (OR = 12.20, 95%CI: 3.34–43.48, *P* = 1.52E-4).

**Table 4:** Logistic regression analysis for independent risk factors of IS.

Variables	B	Walds	OR (95% CI)	*P* value
Hypertension	1.53	93.60	4.62 (3.39–6.30)	3.86E-22
TCH	0.21	4.53	1.24 (1.02–1.50)	0.03
TG	1.81	144.57	6.10 (4.55–8.20)	2.66E-33
LDL-C	0.67	60.33	1.95 (1.65–2.31)	8.00E-15
rs12415607	2.62	15.86	13.67 (3.77–49.48)	6.81E-5
rs2227310	2.50	14.34	12.20 (3.34–43.48)	1.52E-4

IS, ischemic stroke; OR, odds ratio; CI, confidence interval; TCH, total cholesterol; TG, triglyceride; LDL-C, low-density lipoprotein cholesterol.

### The rs12415607 AA genotype associated to lower levels of *CASP7* mRNA

**Figure 1:**
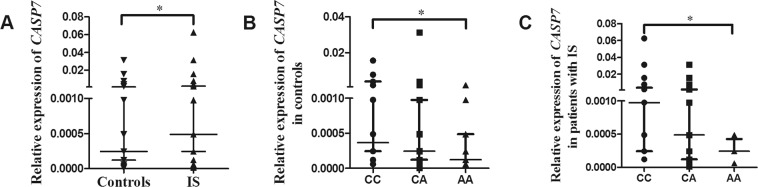
Relative expression of *CASP7* mRNA in IS patients and controls. (**A**) The relative expression of *CASP7* mRNA in IS patients and controls (n = 86); (**B**) The relative expression of *CASP7* mRNA in control subjects carrying the rs12415607 CC, CA, and AA genotype; (**C**) The relative expression of *CASP7* mRNA in IS patients carrying the rs12415607 CC, CA, and AA genotype. Aligned dot plot shows median with interquartile range (*P < 0.05).

**Figure 2:**
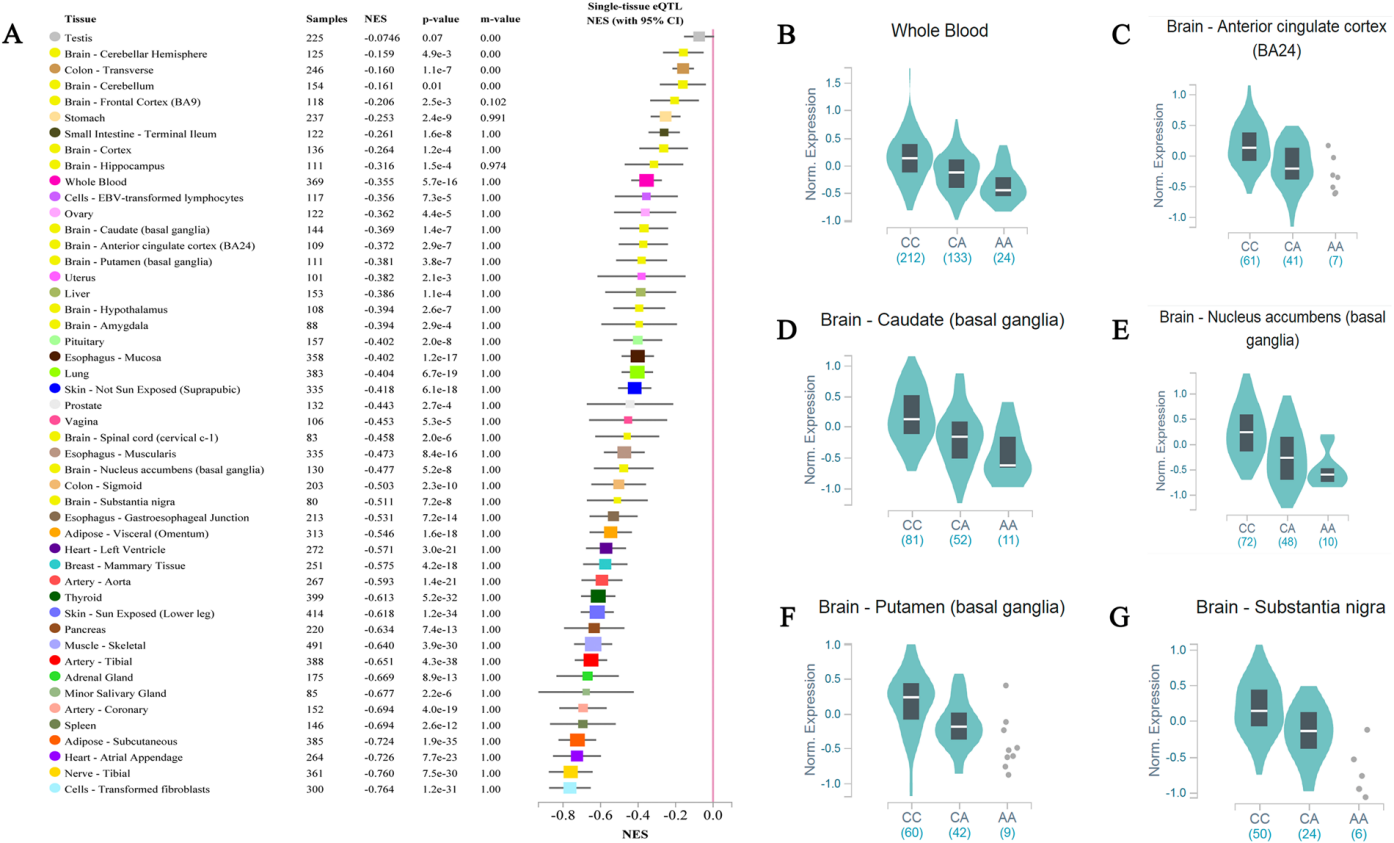
The lower levels of *CASP7* mRNA in the rs12415607 AA carriers were confirmed by eQTL. (**A**) eQTL shows lower levels of *CASP7* mRNA in multiple tissues except for testis; Representative data was presented in single tissue, such as whole blood (**B**), anterior cingulate cortex (**C**), caudate (basal ganglia) (**D**), nucleus accumbens (basal ganglia) (**E**), putamen (basal ganglia) (**F**), and substantia nigra (**G**).

qPCR was used to examine the expression levels of *CASP7* mRNA in IS patients and controls (n = 86). As shown in Fig. 1A, *CASP7* mRNA levels were found to be highly elevated in IS patients compared to controls (*P* = 0.03). We compared *CASP7* mRNA levels in individuals with different genotypes of the rs12415607, and we found that the rs12415607 AA carriers had lower levels of *CASP7* mRNA both in controls (*P* = 0.02, Fig. 1B) and in patients with IS (*P* = 0.03, Fig. 1C). The results were confirmed by data from expression Quantitative Trait Loci (eQTL, https://www.gtexportal.org/home/) which shows lower levels of *CASP7* mRNA in multiple human tissues except for testis (Fig. 2A). Figure 2B–G presents representative data in single tissue, including whole blood, anterior cingulate cortex, caudate (basal ganglia), nucleus accumbens (basal ganglia), putamen (basal ganglia), and substantia nigra.

### The rs12415607 A allele reduced the promoter activity

**Figure 3:**
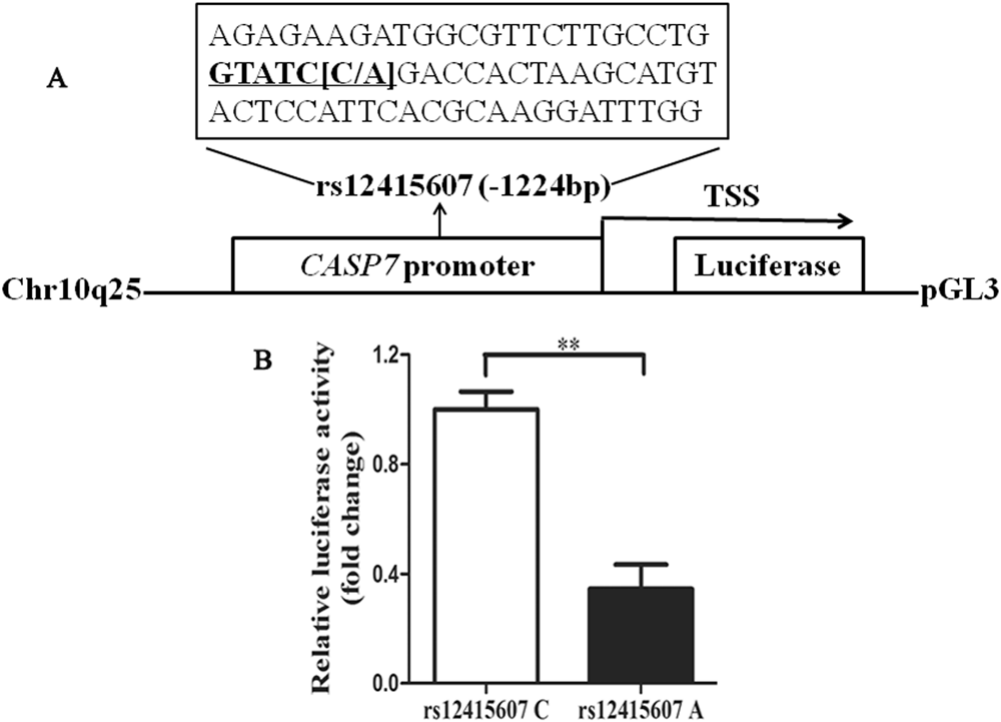
The rs12415607 A allele in the promoter region of *CASP7* reduced the luciferase activity. (**A**) Schematic representation of *CASP7* promoter containing the rs12415607 C/A into pGL3 vector. TSS, transcriptional start site. (**B**) The *CASP7* promoter containing the rs12415607 C or A was inserted into pGL3 vector and transfected into HEK293 cells. At 48 h after transfection, the promoter activity was measured using the Dual Luciferase Reproter assay. Data are presented as mean ± standard error (n = 3, **P < 0.01).

Dual luciferase reporter assay was carried out to assess the effect of the rs12415607 A allele on the promoter activity. Figure 3A shows the schematic representation of *CASP7* promoter containing the rs12415607 C/A into pGL3 vector. Compared to the rs12415607 C, the rs12415607 A exhibited a lower promoter activity (*P* = 0.004, Fig. 3B).

## Discussion

The present study demonstrates that the rs12415607 AA in the promoter of *CASP7* was associated with a reduced risk of IS. Haplotype analysis showed that the CCGAT and AGGAT haplotypes were associated with a reduced risk of IS, whereas the CCGGC, CCGGT, and AGGGT haplotypes were associated with an increased risk of IS. To follow the aim of analyzing the possible reason for the protective effect of the rs12415607 AA on IS risk, qPCR, and dual-luciferase reporter assay were performed, and we found that the rs12415607 AA genotype associated to lower levels of *CASP7* mRNA and reduced promoter activity. These findings indicate that the rs12415607 may be used as a biomarker for the etiology of IS in the Chinese population.

Over the past decades, apoptosis has been demonstrated to contribute to a significant proportion of neuron death following acute brain ischemia^11,12,31–33^. Several biomarkers of apoptosis have been discovered in cerebrospinal fluid and peripheral blood after IS, such as caspase proteases, notably caspase-3-mediated pathways^34–36^. Clinically, elevated caspase-3/7 activity was reported in both acute and late phases of stroke patients^35–37^, and acute caspase-3/7 activation correlated with TNF-α levels, which is an important mediator for IS progression^35,37,38^. Experimental study also showed that *CASP7* mRNA was increased in a rat model of focal cerebral ischemia^28^. Additionally, microRNA-146a down-regulation correlated to neuroprotection in cerebral ischemic injury *in vitro* by targeting pro-apoptotic genes: *CASP7* and Bcl-2-associated transcription factor 1^39^. Blocking the activity of *CASP7* using some traditional Chinese medicines such as aqueous extracts of Lianqiao (Fructus Forsythiae) and Shouwuteng (Caulis Polygoni multiflori) can reduce stroke-inflicted brain damage^40^. These findings provide strong evidence of causal involvement of *CASP7* in stroke, suggesting that targeting physiologic and pharmacologic inhibitors of *CASP7* may be a critical therapeutic strategy for IS.

*CASP7* is located on chromosome 10q25.3 that has been identified to be a susceptibility locus of IS by GWAS^20^. We hypothesized in this study that SNPs in *CASP7* may affect the individual’s susceptibility to IS. Our findings confirmed this hypothesis, and we found that the rs12415607 AA in the promoter of *CASP7* was a protective factor against the occurrence of IS. To date, only three publications investigating the rs12415607 and all of them were involved in cancer. The rs12415607 A allele not only showed a positive association with the risk of cancer but also modulated survival of patients with non-small cell lung cancer treated with platinum-based chemotherapy^41–43^. Moreover, in this study, we provided the first evidence that the rs2227310 represents an independent risk factor of IS besides the rs12415607. The *CASP7* rs2227310 was associated with a potential apoptosis effect in patients with mitochondrial diabetes but not rheumatoid arthritis^44,45^. The conflicting results indicate that the genetic background is different in different human diseases. Even though no significant association between the rs2227310, rs10787498, rs1127687, and rs4353229 in *CASP7* and IS risk was observed in single site analysis, we found in haplotype analysis that the CCGAT and AGGAT haplotypes were associated with a reduced risk of IS, whereas the CCGGC, CCGGT, and AGGGT haplotypes were associated with an increased risk of IS. These findings further support the idea that IS is a complex disease and related to several genetic sites.

*In vitro* study was then performed to explore the possible reason for the rs12415607 AA decreasing IS risk. After transfection into HEK293 cells, the rs12415607 A allele exhibited a lower level of transcriptional activity compared to the rs12415607 C allele. Genotype-phenotype analysis also showed that the rs12415607 AA carriers had lower levels of *CASP7* mRNA. Our findings were verified by data from eQTL, indicating our results are robust. Taken together, we may conclude that the rs12415607 A allele induces lower levels of transcriptional activity and *CASP7* mRNA, and thus is associated with a reduced risk of IS.

There are limitations in the current study: i) As the study subjects comprised only Han Chinese, further studies will be required in different ethnicities. ii) Environmental factors are evident to be responsible for the development of IS^5–8^, gene–environment interaction analysis cannot be performed due to lack of objective data in this study. iii) The study design is hospital-based and selection bias cannot be ruled out, and thus population-based association studies are of great importance.

In conclusion, the present results suggest that the *CASP7* rs12415607 on chromosome 10q25.3 may be a susceptibility locus for IS in Chinese individuals. The protective effect of the rs12415607 AA on IS risk may be explained by decreasing *CASP7* mRNA levels. Determination of the *CASP7* rs12415607 genotype may prove informative for assessment of the genetic risk of IS in such a population. Further studies are warranted to confirm these findings, especially in different ethnic groups.

## Supplementary information

Detailed information of the polymorphisms in the 3′- untranslated region of CASP7 and predicted binding miRNAs
