# Supplementary material for: Association of genetic polymorphisms in CASP7 with risk of ischaemic stroke
Source: Sci Rep. 2019 Dec 9;9:18627. doi: 10.1038/s41598-019-55201-y (PMC6901581; doi:10.1038/s41598-019-55201-y)
Supplement: Supplementary file 1 — Detailed information of the polymorphisms in the 3′- untranslated region of CASP7 and predicted binding miRNAs [file 41598_2019_55201_MOESM1_ESM.pdf]

Association of genetic polymorphisms in *CASP7* with risk of ischaemic stroke

Zhaoshi Zheng [1], Songyan Liu [1], Chuheng Wang [2], Chunhui Wang [3], Dong Tang [1], Yuqing Shi [1], Xuemei Han [1]*

[1] No. 1 Department of Neurology, China-Japan Union Hospital of Jilin University, Changchun, Jilin, 130031, P.R.China

[2] Department of Clinical Medicine (Grade 2017 Student), School of Basic Medicine, Zhengzhou University, Zhengzhou, Henan, 450001, P.R.China

[3] Department of Neurosurgery, the Hospital of Jilin Province, Changchun, Jilin, 130031, P.R.China

Running head: Association of *CASP7* polymorphisms with IS risk

* Corresponding author: Xuemei Han

Tel: +86-0431-84679655

Fax: +86-0431-84679655

xuemeihan1971@163.com

E-mail address:

Zhaoshi Zheng: 325861725@qq.com

Songyan Liu: yan1966@163.com

Chuheng Wang: 1178744265@qq.com

Chunhui Wang: 1409422404@qq.com

Dong Tang: 1391877988@qq.com

Yuqing Shi: syq648356153@163.com

Xuemei Han: xuemeihan1971@163.com

Supplementary Table 1 Detailed information of the polymorphisms in the 3’-untranslated region of *CASP7* and predicted binding miRNAs

Polymorphisms	Predicted binding miRNAs
rs10787498	hsa-miR-129-5p
	hsa-miR-130a-3p
	hsa-miR-130b-3p
	hsa-miR-140-5p
	hsa-miR-19a-3p
	hsa-miR-19b-3p
	hsa-miR-301a-3p
	hsa-miR-301b
	hsa-miR-3666
	hsa-miR-4295
	hsa-miR-4480
	hsa-miR-454-3p
	hsa-miR-4643
	hsa-miR-519a-3p
	hsa-miR-519b-3p
	hsa-miR-519c-3p
rs1127687	hsa-miR-140-5p
	hsa-miR-4264
	hsa-miR-4697-3p
	hsa-miR-5693
rs4353229	hsa-miR-224-5p
	hsa-miR-4257
	hsa-miR-520a-5p
	hsa-miR-525-5p
